# Supplementary material for: CD103–CD8+ T cells promote neurotoxic inflammation in Alzheimer’s disease via granzyme K–PAR-1 signaling
Source: Nat Commun. 2025 Sep 24;16:8372. doi: 10.1038/s41467-025-62405-6 (PMC12460627; doi:10.1038/s41467-025-62405-6)
Supplement: Supplementary file 1 — Supplementary Information [file 41467_2025_62405_MOESM1_ESM.pdf]

CD103⁻CD8⁺ T cells promote neurotoxic inflammation in Alzheimer's disease via granzyme K–PAR-1 signaling

Eleonora Terrabuio[1,2*], Enrica Caterina Pietronigro[1], Alessandro Bani[1], Vittorina Della Bianca[1], Carlo Laudanna[1,2], Barbara Rossi[1], Giulia Finotti[3], Bruno Santos-Lima[1], Elena Zenaro[1], Ermanna Turano[4], Gabriele Tosadori[1], Matteo Calgaro[5], Nicola Vitulo[5], Monica Castellucci[3], Daniela Cecconi[5], Jessica Brandi[5], Nikolaos Vareltzakis[1], Fabiana Mainieri[1], Antonella Calore[1], Gabriele Angelini[1], Bruno Bonetti[6], Gabriela Constantin[1, 2,*]

[1]Department of Medicine, University of Verona, Strada le Grazie 8, 37134 Verona, Italy

[2]The Center for Biomedical Computing (CBMC), University of Verona, 37134 Verona, Italy

[3]Centro Piattaforme Tencologiche (CPT), University of Verona, 37134 Verona, Italy

[4]Department of Neuroscience, Biomedicine and Movement Sciences, University of Verona, 37134 Verona, Italy

[5]Department of Biotechnology, University of Verona, Strada Le Grazie 15, 37134 Verona, Italy

[6]Neurology Unit, Azienda Ospedaliera Universitaria Integrata of Verona, P. le Stefani 37138 Verona, Italy

*Correspondence and requests for materials should be addressed to Gabriela Constantin (gabriela.constantin@univr.it) or Eleonora Terrabuio (eleonora.terrabuio@univr.it)

Supplementary Figure 1

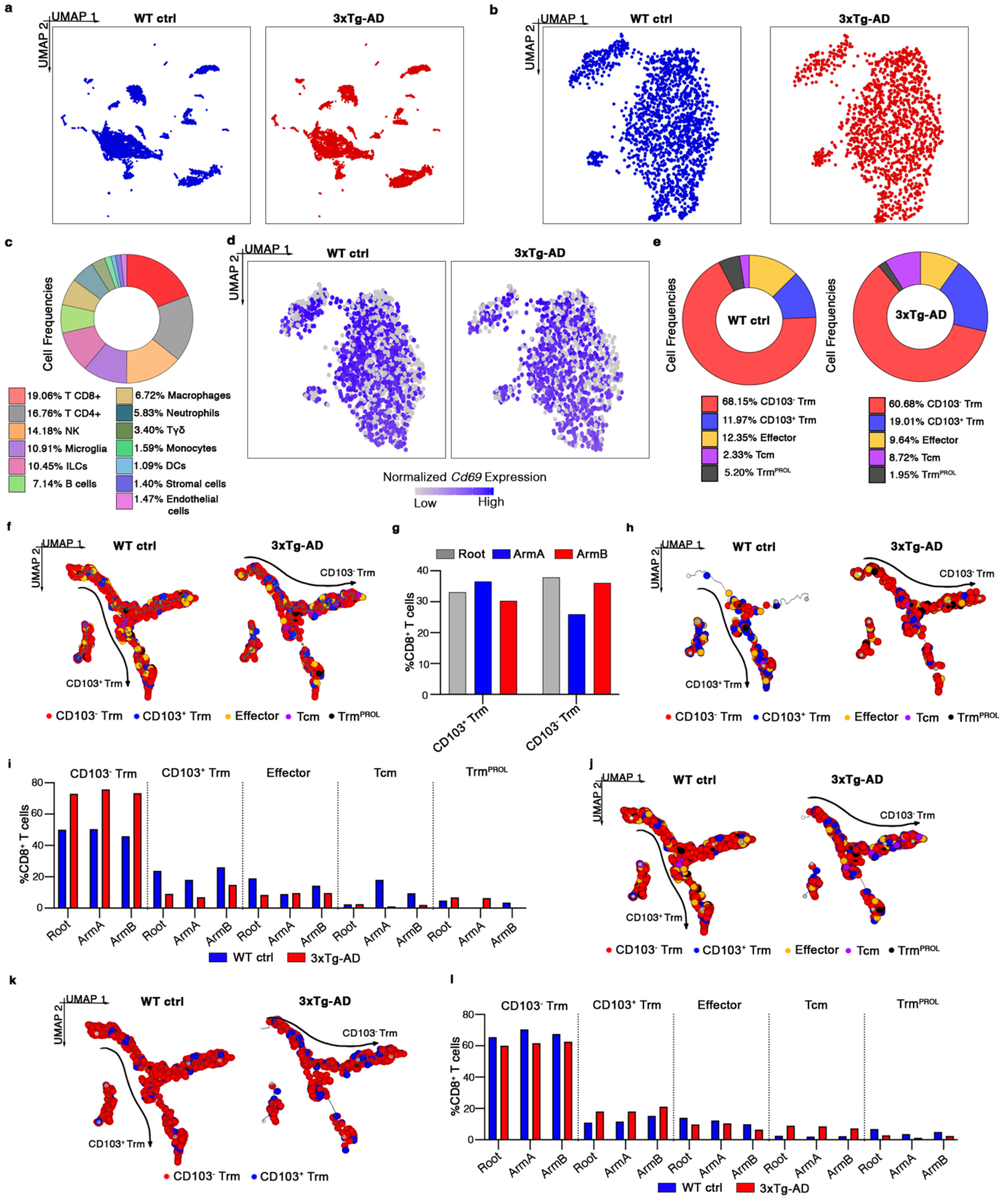

a
WT ctrl
3xTg-AD
UMAP 1
UMAP 2
b
c
Cell Frequencies
19.06% T CD8+
16.76% T CD4+
14.18% NK
10.91% Microglia
10.45% ILCs
7.14% B cells
6.72% Macrophages
5.83% Neutrophils
3.40% Tγδ
1.59% Monocytes
1.09% DCs
1.40% Stromal cells
1.47% Endothelial cells
d
Normalized Cd69 Expression
Low
High
e
68.15% CD103- Trm
11.97% CD103+ Trm
12.35% Effector
2.33% Tcm
5.20% TrmPROL
60.68% CD103- Trm
19.01% CD103+ Trm
9.64% Effector
8.72% Tcm
1.95% TrmPROL
f
CD103- Trm
CD103+ Trm
Effector
Tcm
TrmPROL
g
Root
ArmA
ArmB
%CD8+ T cells
h
i
j
k
l
WT ctrl
3xTg-AD

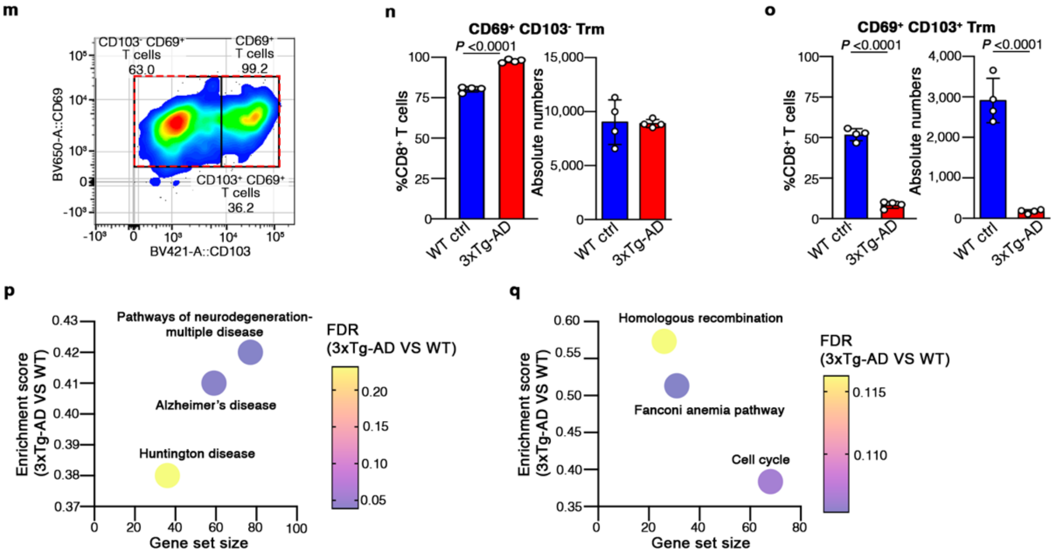

Supplementary Fig. 1. Distribution of the CD8$^+$ T cell compartment in the brains and meninges of AD mice. a, UMAP plot showing CD45^{HIGH} leukocytes detected in the brains and meninges of wild-type control (WT; n = 8) and 3xTg-AD (n = 8) mice (6 months old). **b**, UMAP plot showing CD8$^+$ T cells detected in the brains and meninges of WT (n = 8) and 3xTg-AD (n = 8) mice (6 months old). **c**, Donut plot showing the frequencies of the different cell populations among all CD45^{HIGH} leukocytes detected in the brains and meninges of WT (n = 8) and 3xTg-AD (n = 8) mice (6 months old). **d**, UMAP plot showing normalized *Cd69* expression in CD8$^+$ T cells in the brains and meninges of WT (n = 8) and 3xTg-AD (n = 8) mice. Transcript levels are color coded: violet high expression, white low expression. **e**, Donut plots indicating the distribution of CD8$^+$ T cell subsets in the meninges of WT (left) and 3xTg-AD (right) mice. **f**, Trajectory plots indicating the distribution of CD8$^+$ T cell subsets in the brains and meninges of WT (left) and 3xTg-AD (right) mice. **g**, Bar plots showing the percentage of CD103$^+$ and CD103$^-$ Trm CD8$^+$ T cell subsets in the brains and meninges of WT and 3xTg-AD mice distributed in the root, arm A and arm B of the trajectory plot. **h**, Trajectory plots indicating the distribution of CD8$^+$ T cell subsets in the brains of WT (left) and 3xTg-AD (right) mice. **i**, Bar plots showing the percentage of each CD8$^+$ T cell subset in the root, arm A, and arm B of the trajectory plot representing the brains of WT and 3xTg-AD mice. Arms A and B are characterized by *Itgae* and *Eomes* gene expression, respectively. **j**, Trajectory plots indicating the distribution of CD8$^+$ T cell subsets in the meninges of WT (left) and 3xTg-AD (right) mice. **k**, Trajectory plots indicating the distribution of CD103$^-$ and CD103$^+$ CD8$^+$ Trm cell subsets in the meninges of WT (left) and 3xTg-AD (right) mice. **l**, Bar plots showing the percentage of each CD8$^+$ T cell subset in the root, arm A, and arm B of the trajectory plot representing the meninges of WT and 3xTg-AD mice. Arms A and B are characterized by *Itgae* and *Eomes* gene expression, respectively. **m,** Gating strategy showing the percentage of CD69$^+$ CD8$^+$ Trm cells (red gate) in

mouse brains. **n, o**, Percentages (left) and absolute numbers (right) of $CD103^{-}$ (n) and $CD103^{+}$ (o) $CD69^{+}CD8^{+}$ Trm cells in the meninges of WT (n = 4) and 3xTg-AD (n = 4) mice (6 months old). Data are means ± SD, with *P*-values based on Student's t-test. **p, q**, GSEA applied to the KEGG database showing the three most enriched terms in the brains (n) and meninges (o) of 3xTg-AD mice compared to WT controls. Dot color = false discovery rate (FDR).

Supplementary Figure 2

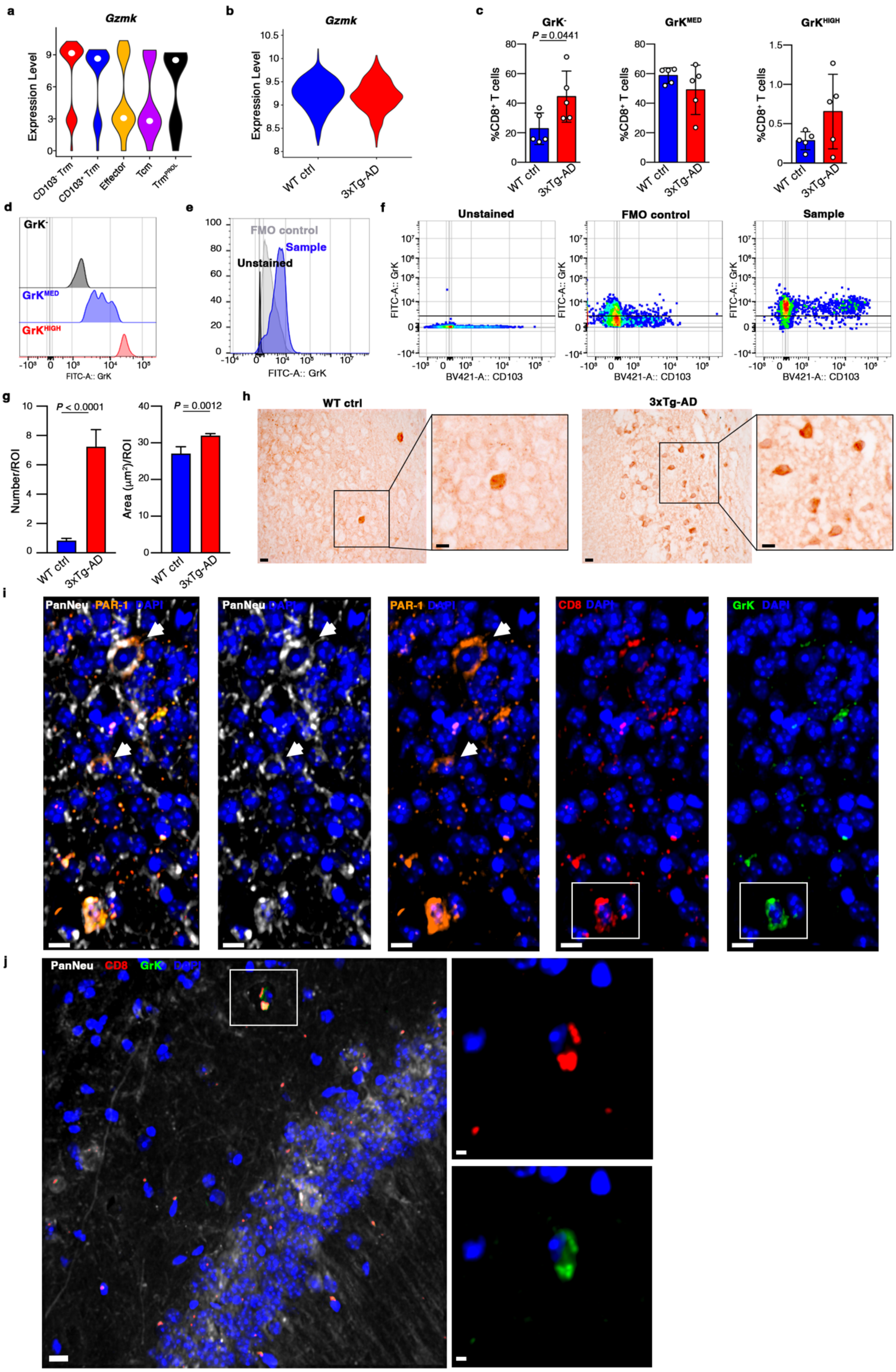
a
Gzmk
Expression Level
CD103⁻ Trm
CD103⁺ Trm
Effector
Tcm
Trm^PROL
b
Gzmk
Expression Level
WT ctrl
3xTg-AD
c
GrK⁻
P = 0.0441
GrK^MED
GrK^HIGH
%CD8⁺ T cells
d
GrK⁻
GrK^MED
GrK^HIGH
FITC-A:: GrK
e
FMO control
Sample
Unstained
FITC-A:: GrK
f
Unstained
FMO control
Sample
FITC-A:: GrK
BV421-A:: CD103
g
P < 0.0001
P = 0.0012
Number/ROI
Area (µm²)/ROI
h
WT ctrl
3xTg-AD
i
PanNeu PAR-1 DAPI
PanNeu DAPI
PAR-1 DAPI
CD8 DAPI
GrK DAPI
j
PanNeu CD8 GrK DAPI

Supplementary Fig. 2. *Gzmk* expression in meningeal CD103⁻ CD8⁺ Trm cells. a, Violin plot showing the expression of *Gzmk* in $CD8^+$ T cell subsets in our dataset (WT and 3xTg-AD mice; brains and meninges). **b**, Violin plot showing the expression of *Gzmk* in $CD8^+$ T cells in the meninges of WT and 3xTg-AD mice. **c,** Bar plots showing the percentage of GrK^-CD103^-, $GrK^{MED}CD103^-$, and $GrK^{HIGH}CD103^-$ $CD8^+$ Trm cells in the meninges of WT (n = 5) and 3xTg-AD (n = 5) mice (6 months old). Data are means ± SD, with *P*-values based on Student's t-test. **d**, Representative histograms showing GrK levels in GrK^- (black), GrK^{MED} (blue) and GrK^{HIGH} (red) cell populations detected by flow cytometry. **e, f,** Histograms (e) and pseudocolor plots (f) showing GrK levels in unstained control (black), FMO control (gray), and sample (blue). **g, h**, Immunohistochemical analysis (g) in the hippocampus of WT and 3xTg-AD mice evaluating the number (left) and the area (right) of PAR-1^+ neurons, with representative images (h). Scale bar = 20 µm, or 10 µm in the zoomed images. Data are means ± SD, with *P*-values based on Student's t-test. **i**, $CD8^+GrK^+$ cells (squares) near PAR-1^+ neurons (arrows) in the hippocampus of 3xTg-AD mice (6 months old) detected by immunofluorescence staining (CD8 = red, PanNeu = white, GrK = green, PAR-1 = orange) with DAPI nuclear counterstaining (blue). Scale bar = 5 µm. **j**, $CD8^+$ T cell (square) in the brains of WT mice (6 months old) detected by immunofluorescence staining (CD8 = red, PanNeu = white, GrK = green) with DAPI nuclear counterstaining (blue). Scale bar = 5 µm or 2 µm for zoomed images.

Supplementary Figure 3

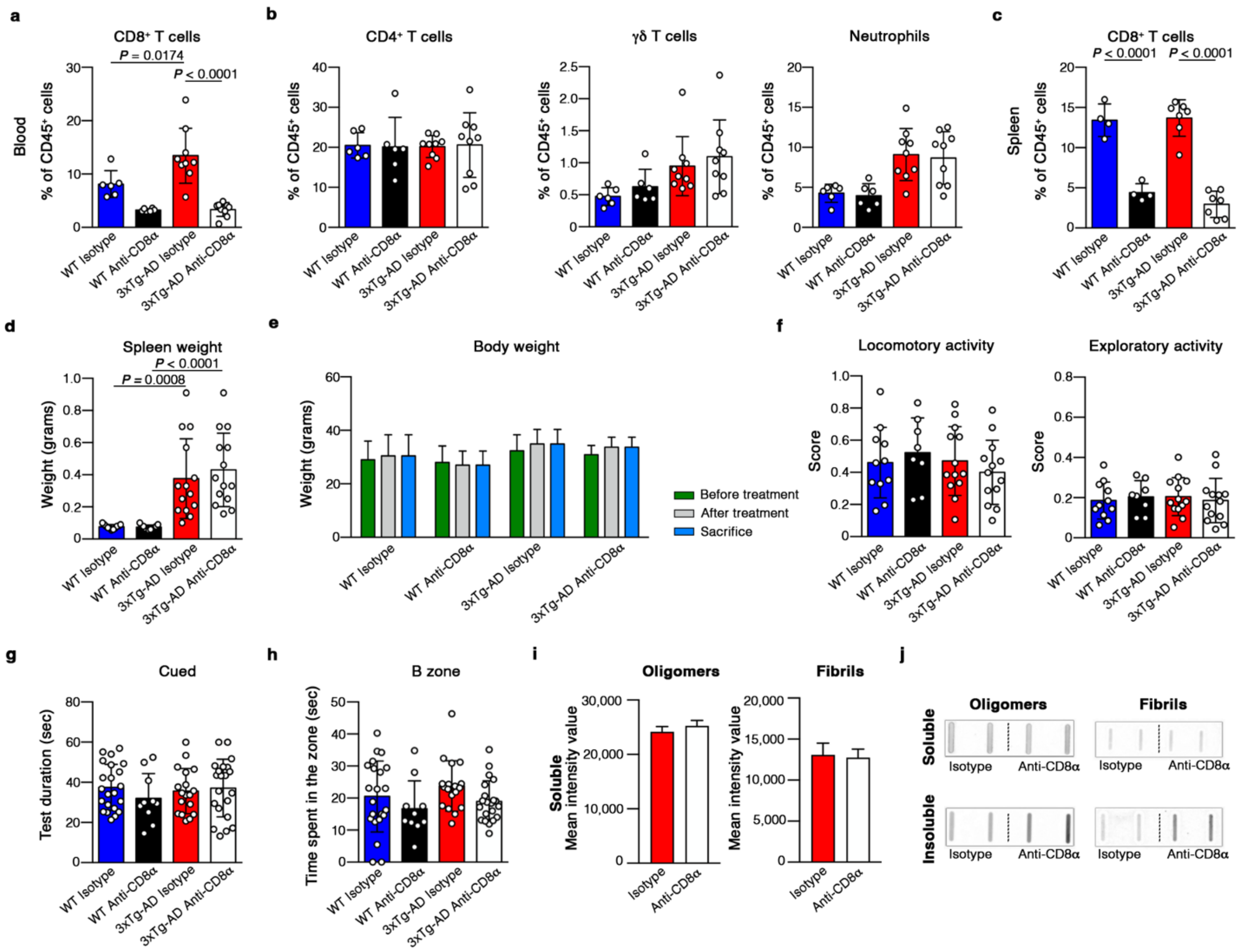

Supplementary Fig. 3. Anti-CD8α treatment successfully depletes peripheral CD8$^+$ T cells. a, **b,** Bar plots showing the percentage of CD8$^+$ T cells (a), CD4$^+$, γδ T cells and neutrophils (b) in the blood of WT isotype control (blue, n = 6), CD8-depleted WT (black, n = 6), 3xTg-AD isotype control (red, n = 9) and CD8-depleted 3xTg-AD (white , n = 9) mice. Data are means ± SD, with *P*-values based on one-way ANOVA multiple comparisons. **c**, Bar plot showing the percentage of CD8$^+$ T cells in the spleens of isotype control and CD8-depleted WT and 3xTg-AD mice (colors, sample numbers and statistical testing as above). **d**, Spleen weight of isotype control and CD8-depleted WT and 3xTg-AD mice (colors, sample numbers and statistical testing as above). **e**, Body weight of isotype control and CD8-depleted WT and 3xTg-AD mice. Data show mice before (green) and after (gray) the treatment, and before euthanasia (light blue). **f**, Open field behavioral test showing no differences in locomotor activity (left) and exploratory behavior (right) in WT isotype control (blue, n = 11), CD8-depleted WT (black, n = 8), 3xTg-AD isotype control (red, n = 14) and CD8-depleted 3xTg-AD (white, n = 13) mice. Data are means ± SD. **g**, Bar plot showing the time taken (in seconds) by WT isotype control (blue, n = 22), CD8-depleted WT (black, n = 10), 3xTg-AD isotype control

(red, n = 18) and CD8-depleted 3xTg-AD (white, n = 21) mice to reach the platform during the cued stage of the MWM behavioral test. Data are means ± SD based on two independent experiments. **h**, Bar plot showing the time spent (in seconds) in the target zone by isotype control and CD8-depleted WT and 3xTg-AD mice (colors, sample numbers and statistical testing as in panel (g)) during the probe stage of MWM behavioral test. **i,** Dot blot experiments showing the amount of soluble oligomeric (A11 antibody, left) and fibrillar (OC antibody, right) forms of Aβ in brain homogenates from 3xTg-AD mice treated with an anti-CD8 antibody (n = 4) or an isotype control (n = 4). Data from three independent experiments are shown as means ± SEM. **j**, Representative images of dot blot experiments performed on soluble (upper panels) and insoluble (bottom panels) fractions of brain homogenates from 3xTg-AD mice treated with an anti-CD8 antibody or an isotype control, evaluating Aβ oligomers (A11 antibody, left) and fibrils (OC antibody, right). Uncropped dot blots are provided in Supp. Data 5-8.

Supplementary Figure 4

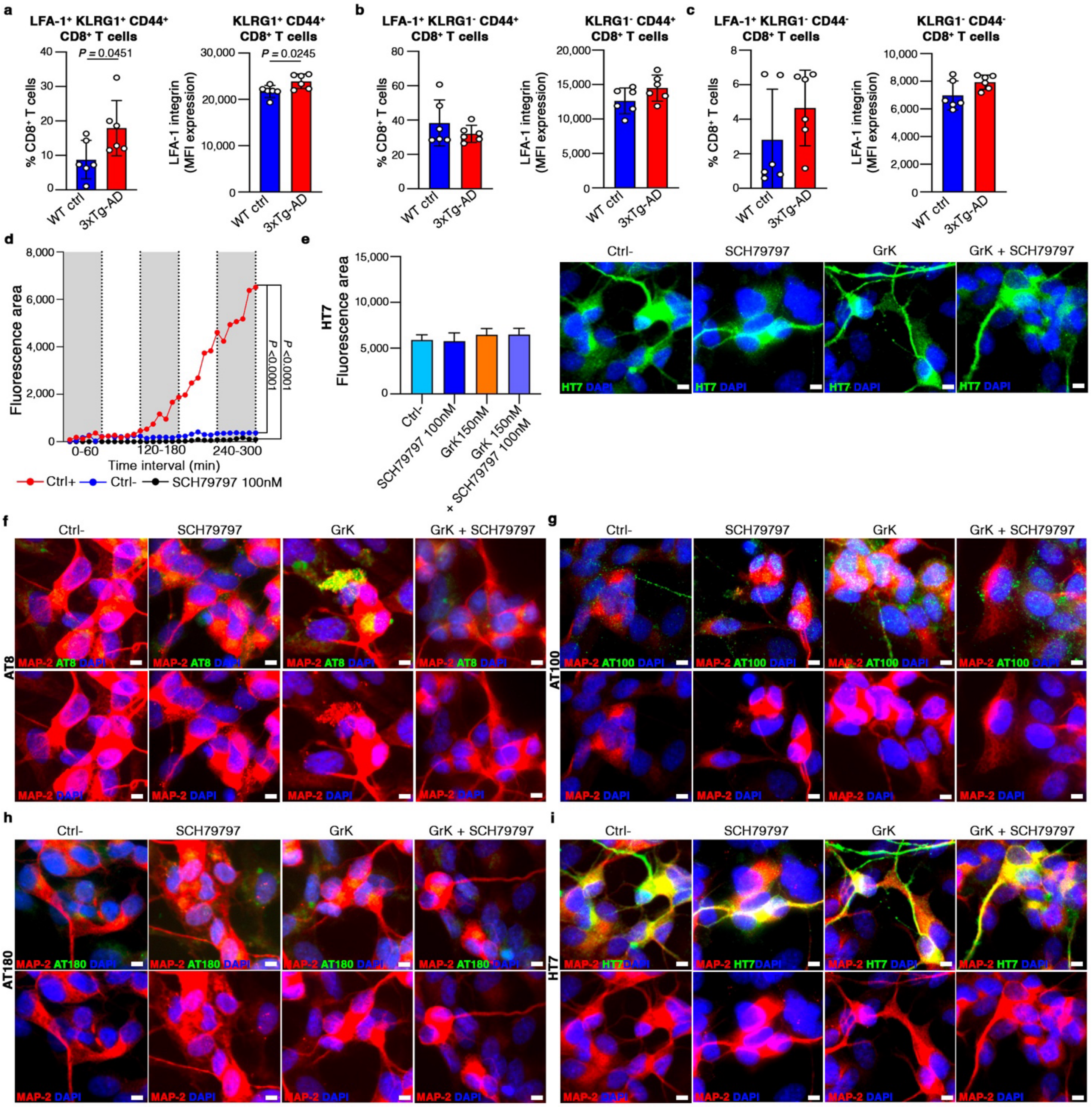

Supplementary Fig. 4. Expression of LFA-1 integrin on circulating CD8$^+$ T cells in AD mice. a-c, Bar plots showing the percentage of LFA-1$^+$ cells (left) and the LFA-1 mean fluorescence intensity (MFI, right) for KLRG1$^+$CD44$^+$ (a), KLRG1$^-$CD44$^+$ (b), and KLRG1$^-$CD44$^-$ (c) CD8$^+$ T cells in the blood of WT (n = 6) and 3×Tg-AD (n = 6) mice (6 months old). Data are means ± SD, with *P*-values based on Student's t-test. **d**, Intracellular Ca^{2+} release in differentiated SH-SY5Y cells cultured for 5 hours in the presence of 100 nM of the PAR-1 inhibitor SCH79797 (violet) to demonstrate that SCH79797 alone does not affect neuronal functionality. Ctrl$^-$ (blue) = neurons alone. Ctrl$^+$ (red) = ionomycin-stimulated neurons (10 μM). Data are means ± SD based on two independent experiments,

with *P*-values based on two-way ANOVA multiple comparisons. **e**, Bar plots (left) and representative images (right) showing levels of total tau (HT7) on differentiated SH-SY5Y cells cultured in the absence of active GrK (Ctrl⁻), in the presence of 100 nM SCH79797 alone, in the presence of 150 nM active GrK alone, and in the presence of both. Data are means ± SD based on three independent experiments, with *P*-values based on two-way ANOVA multiple comparisons. Nuclei were counterstained with DAPI. Scale bar = 5 μm. **f-i**, Representative images showing the expression of MAP-2 (red) and AT8 (f), AT100 (g), AT180 (h), and HT7 (i) (green) in differentiated SH-SY5Y cells cultured in the absence of active GrK (Ctrl⁻), in the presence of 100 nM SCH79797 alone, in the presence of 150 nM active GrK alone, and in the presence of both (data from three independent experiments). Nuclei were counterstained with DAPI. Scale bar = 5 μm.

Supplementary Figure 5

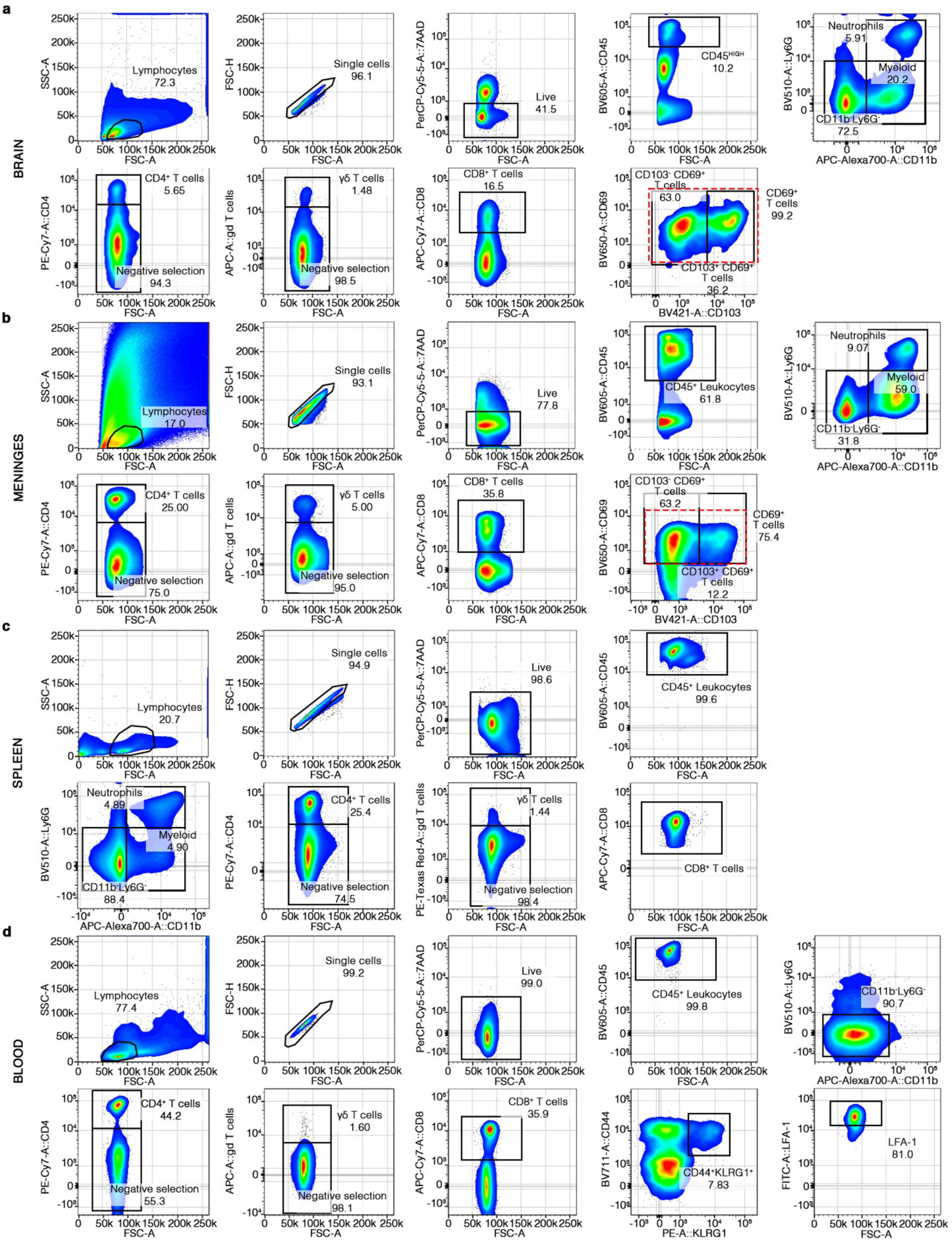

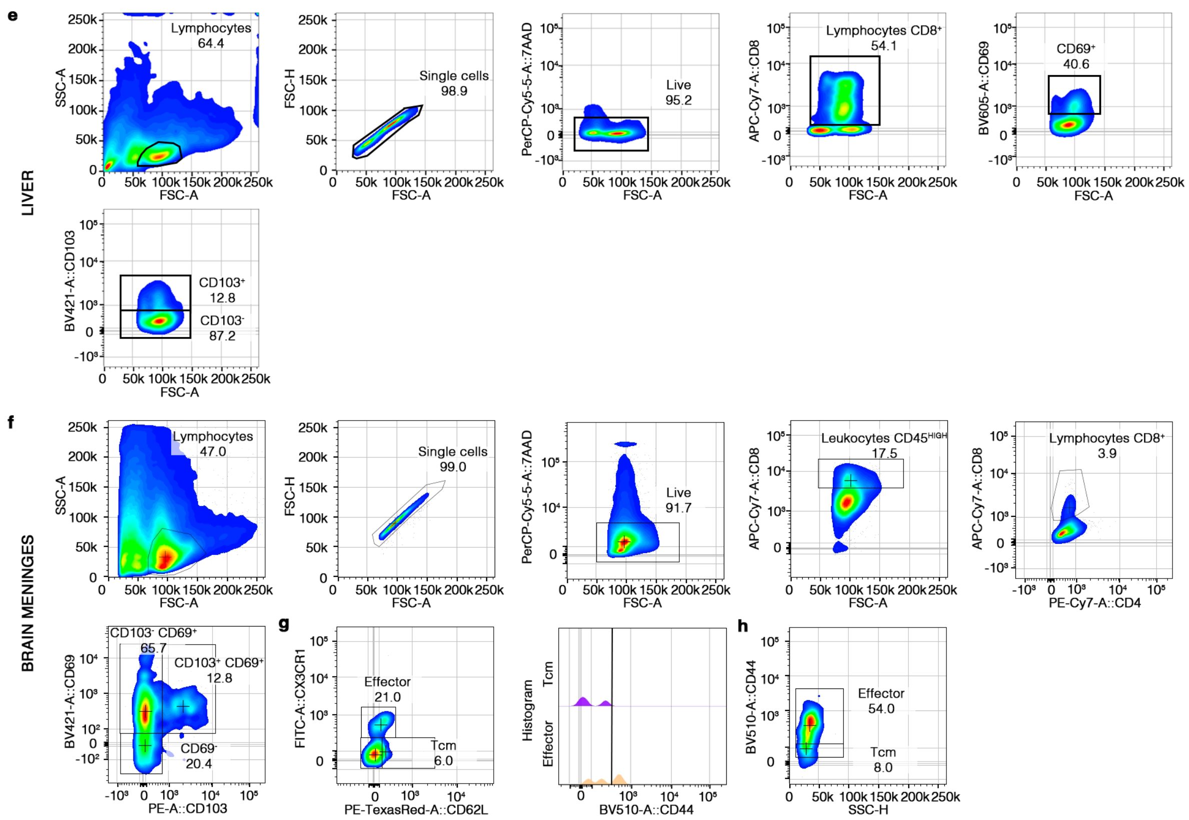

Supplementary Fig. 5. Gating strategies for flow cytometry data and cell sorting. a-d, Dot plots showing gating strategies used to analyze brain (a), meninges (b), spleen (c) and blood (d) samples. $CD69^{+}CD8^{+}$ T cells in the brain (a) and meninges (b) are gated in red. Percentages of $CD103^{-}$ Trm and $CD69^{+}$ Trm cell populations were calculated based on the $CD8^{+}$ T cell population. Percentages shown in the plots are calculated based on the parent gate. **e**, Gating strategy used to sort $CD69^{+}CD103^{–}$ and $CD69^{+}CD103^{+}$ $CD8^{+}$ Trm cells from the livers of 3xTg-AD mice (6 months old) for *in vitro* experiments. Percentages shown in the plots are calculated based on the parent gate. **f-h**, Gating strategies used to analyze brain and meninges samples calculating the expressions of intracellular and extracellular phenotypical markers of $CD103^{-}CD69^{+}$ Trm, $CD103^{+}CD69^{+}$ Trm, $CD69^{-}$ Effector and $CD69^{-}$ Tcm $CD8^{+}$ T cell subsets. Effector and Tcm populations were gated starting from the $CD69^{-}$ subset of $CD8^{+}$ T cells. For the extracellular panel (g), Effector and Tcm populations were identified as $CD44^{HIGH}CX3CR1^{+}CD62L^{-}$ and $CD44^{LOW}CX3CR1^{-}CD62L^{+}$, respectively. For the intracellular panel (h), Effector and Tcm populations were identified as $CD44^{HIGH}$ or $CD44^{LOW}$, respectively. The MFI of CD69, CD103, and CX3CR1 for the extracellular panel (g) and of EOMES, GrK, GrB, and GrA for the intracellular panel (h) was calculated for each cell population. Percentages shown in the plots are calculated based on the parent gate.

Supplementary Figure 6

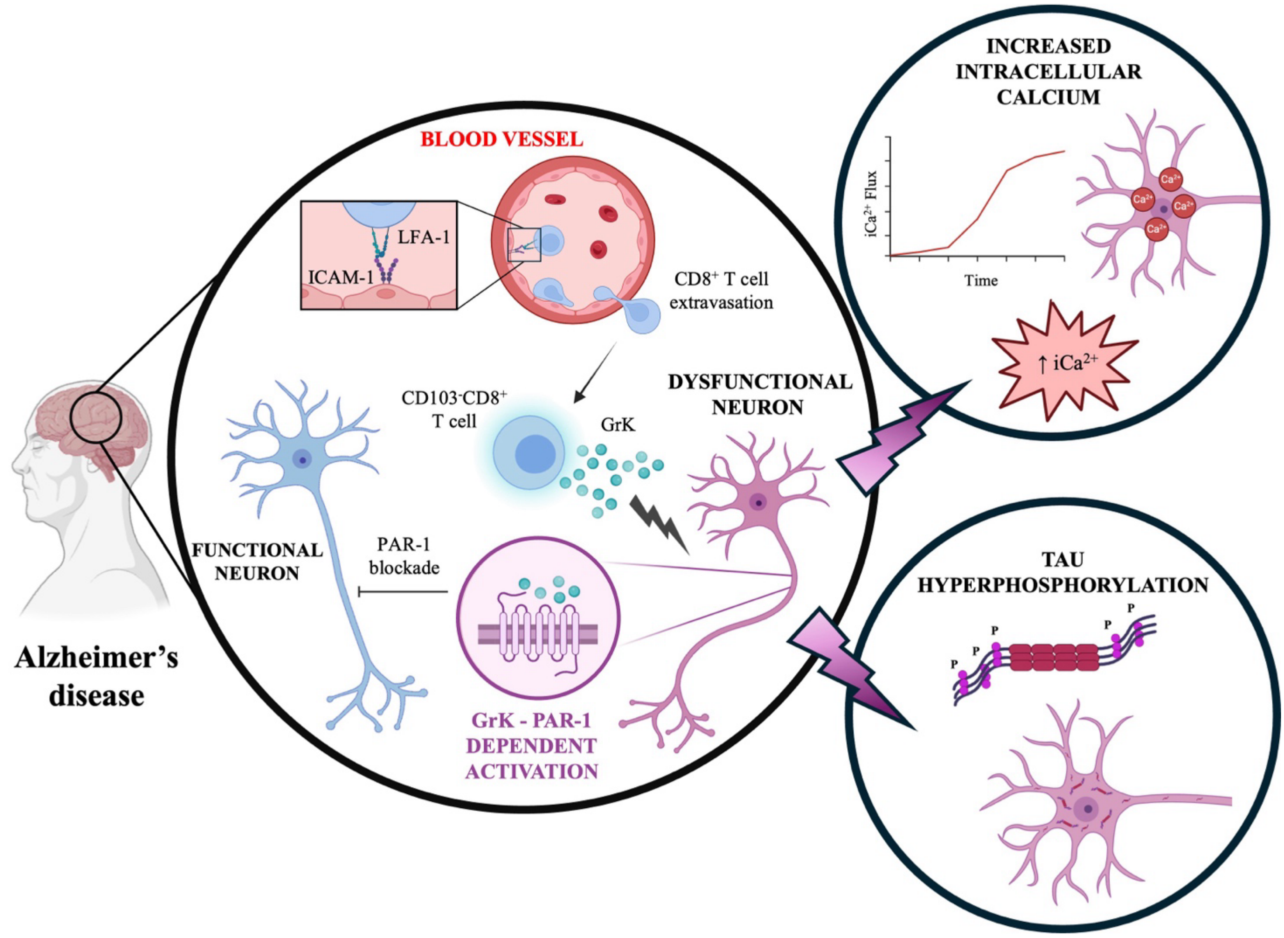

Supplementary Fig. 6. GrK$^+$CD103$^-$CD8$^+$ Trm cells mediate neurotoxic inflammation in AD. CD103$^-$CD8$^+$ Trm cells accumulate in the brains of AD animal models and human AD patients. These cells are replenished from the blood and require LFA-1 integrin for extravasation into the brain. Depletion of brain CD103$^+$CD8$^+$ T cells ameliorates the cognitive deficit and reduces neuropathological changes in AD mice. Inside the brain parenchyma, CD103$^-$CD8$^+$ Trm cells produce large amounts of GrK, directly activating neuronal PAR-1. The GrK–PAR-1 axis then induces tau hyperphosphorylation and neuronal dysfunction. GrK-induced neuronal alterations are prevented by PAR-1 blockade. Collectively, our data show that dysfunctional communication between the immune system and CNS mediated by CD103$^-$CD8$^+$ T cells and GrK–PAR-1 signaling contributes to the development of AD, identifying new molecular mechanisms that can be targeted to prevent immune-mediated neurotoxic inflammation. Created in BioRender. Terrabuio, E. (2025) https://BioRender.com/fllxi6t.

Supplementary Tables

Supplementary Table 1 | Clinical data of human post-mortem hippocampus specimens used for the analysis of intraparenchymal and intravascular $CD103^-$, $CD103^+$, and GrK^+ $CD103^-$ $CD8^+$ T cells.

ID	Neuropathology	Age	Sex
CTRL			
A308/14	Minimal ageing changes BNE stage, control	66	F
A012/12	Normal brain	51	F
A250/14	Control brain	69	F
AD			
A038/17	AD BNE stage 6, MAA	69	F
A226/16	AD BNE stage 6	69	F
A272/16	AD BNE stage 6	68	F

Detailed neuropathology of each individual, included in the study, along with age and sex. The average age is 62 ± 9.2 (mean ± standard deviation) for controls (CTRL) (*n* = 3) and 68.7 ± 0.6 (mean ± standard deviation) for AD individual (*n* = 3). AD = Alzheimer's disease; BNE = Brain Net Europe; MAA=Mild Amyloid Angiopathy.

Supplementary Table 2 | List of reagents

Reagents	Assay	Source	Identifier
7-AAD Viability Staining Solution	FC	BioLegend	Cat # 420404
Anti-CD16/32 Fc-Block	FC	BioLegend	Cat # 101302
Anti-CD11a/CD18 FITC	FC	Miltenyi Biotech	Cat # 130-114-422
Anti-CD103 BV421	FC	BD Bioscences	Cat # 562771
Anti-CD8 APC-H7	FC	BD Bioscences	Cat # 560182
Anti-CD69 BV650	FC	BD Bioscences	Cat # 740460
Anti-TCRgd PE-CF594	FC	BD Bioscences	Cat # 563532
Anti-CD62L PE	FC	BD Bioscences	Cat # 553151
Anti-CD45 BV605	FC	BD Bioscences	Cat # 563053
Anti-CD27 APC	FC	BD Bioscences	Cat # 560961
Anti-CD11b APC-R700	FC	BD Bioscences	Cat # 564985
Anti-Ly6g BV510	FC	BD Bioscences	Cat # 740157
Anti-CD4 PE-Cy7	FC	BD Bioscences	Cat # 552775
Anti-CD197 (CCR7) BV786	FC	BD Bioscences	Cat # 564355
Anti-CD44 BV711	FC	BD Bioscences	Cat # 563971
Viobility TM Fixable Dye 405/520	FC	Miltenyi Biotech	Cat # 130-109-814
Anti-CD45 APC-Vio770	FC	Miltenyi Biotech	Cat # 130-110-662
Anti-CD8 PE-Cy7	FC	Biolegend	Cat # 100722
Anti-CD3 BV650	FC	BD Bioscences	Cat # 564378
Rabbit anti-mouse/human GrK	FC/IF	ThermoFisher Scientific	Cat # PA550980
Goat anti-rabbit AlexaFluor 488	FC/IF	ThermoFisher Scientific	Cat # A11034
Anti-VLA-4 (CD49d) PE-Cy7	FC	BioLegend	Cat # 103618
Anti-Ly6g BV421	FC	BD Bioscences	Cat # 562737
Anti-CD44 BV510	FC	BD Bioscences	Cat # 563114
Anti-CD45 BV786	FC	BD Bioscences	Cat # 564225
Anti-CD4 APC	FC	BD Bioscences	Cat # 553051
Anti-CD45 BV480	FC	BD Bioscences	Cat # 566095
Anti-KLRG1	FC	BD Bioscences	Cat # 561621
Anti-GrA eFluor450	FC	ThermoFisher Scientific	Cat # 48-5831-82
Anti-CD69 BV605	FC	BD Bioscences	Cat # 563290
Anti-TCRgd BV650	FC	ThermoFisher Scientific	Cat # 416-5711-82
Anti-CD103 BV711	FC	ThermoFisher Scientific	Cat # 407-1031-82
Anti-CD45 BV786	FC	ThermoFisher Scientific	Cat # 417-0451-82
Anti-GrB PE	FC	SONY	Cat # 2461035
Anti-CD4 Pe-Cy7	FC	ThermoFisher Scientific	Cat # 25-0041-82
Anti-Eomes eFluor660	FC	ThermoFisher Scientific	Cat # 50-4875-82
Fixable dye 700	FC	BD Biosciences	Cat # 564997
Anti-CD8 APC-AF750	FC	ThermoFisher Scientific	Cat # MCD0827
Anti-CD69 SB436	FC	ThermoFisher Scientific	Cat # 62-0691-82
Anti-CXCR3	FC	ThermoFisher Scientific	Cat # 63-1831-82
Anti-CD45 BV711	FC	ThermoFisher Scientific	Cat # 407-0451-82
Anti-CX3CR1 FITC	FC	ThermoFisher Scientific	Cat # ACR-059-F
Anti-CD103 PE	FC	ThermoFisher Scientific	Cat # 12-1031-82
Anti-CD62L PE-TexasRed	FC	ThermoFisher Scientific	Cat # RM4317
Anti-CXCR6 APC	FC	ThermoFisher Scientific	Cat # 17-9186-82

Fixation Buffer	FC	BD Bioscences	Cat # 55655
Staining Perm Wash Buffer	FC	BD Bioscences	Cat # 554723
Transcription factor buffer set	FC	BD Pharmigen	Cat # 562574
Collagenase crude type IA	FC	Merck Millipore	Cat # C2674-100MG
Deoxiribonuclease I crude lyophilized	FC	Merck Millipore	Cat # DN25-100MG
Recombinant Mouse IL-2 Protein	In Vitro cultures	R&D Systems	Cat # 402-ML
Recombinant Mouse IL-7 Protein	In Vitro cultures	R&D Systems	Cat # 407-ML-025
Neurobasal medium<br>DMEM/F-12	In Vitro cultures<br>In Vitro cultures	Gibco<br>Biowest	Cat # 21103049<br>Cat # L0090-500
Corning® 500 mL RPMI 1640 1X	Cell isolation/ In Vitro cultures	Corning	Cat # 15-040-CV
B-27 Plus Supplement (50X)	In Vitro cultures	Gibco	Cat # 17504044
Glutagro supplement	In Vitro cultures	Corning	Cat # 25-015-CI
Penicillin-Streptomycin	In Vitro cultures	Sigma-Aldrich	Cat # P4333
Poly-D-lysine hydrobromide	In Vitro cultures	Sigma-Aldrich	Cat # P6407
Laminin	In Vitro cultures	Merck Millipore	Cat # 11243217001
Ionomycin calcium salt from Streptomyces conglobatus	In Vitro cultures	Sigma-Aldrich	Cat # I0634
Human recombinant GrK	In Vitro cultures	Cusabio	Cat # CSB-EP010084HU
Mouse recombinant GrK	In Vitro cultures	Cusabio	Cat # CSB-EP010084MO
Recombinant Human/Murine/Rat BDNF	In Vitro cultures	Peprotech	Cat # 450-02-10UG
N6,2'-O-Dibutirril-adenosina 3',5'-ciclica monofosfato	In Vitro cultures	Sigma-Aldrich	Cat # D0627
SCH79797 PAR-1 inhibitor<br>Reinoic acid	In Vitro cultures<br>In Vitro cultures	DBA<br>Sigma-Aldrich	Cat # Hy-14993<br>Cat # R2625-50MG
Biotracker 609 Red Ca2+ AM Dye<br>CellTracker TM Blue CMAC Dye	Live Imaging<br>Live Imaging	Merck Millipore<br>ThermoFisher Scientific	Cat # SCT021<br>Cat # C34552
Anti-Ras isotype antibody	In Vivo Treatment	Produced in house (Clone Y13-259)	Produced in house
Anti-mouse CD8α	In Vivo Treatment	BioXCell	BE0061
Normal goat serum	IHC/IF	Vector Labs	Cat # S1000
Bovine Serum Albumin	IHC/IF	Sigma-Aldrich	Cat # A4503
Anti-β-Amyloid, 1-16 Antibody (6E10)	IHC	BioLegend	Cat# SIG-39320
Tau Monoclonal Antibody (HT7)	IHC/IF	ThermoFisher Scientific	Cat # MN1000
Phospho-Tau (Thr231) Monoclonal Antibody (AT180)	IHC/IF	ThermoFisher Scientific	Cat # MN1040
Triton™ X-100	IHC	Sigma-Aldrich	Cat # X-100
Hydrogen peroxide solution	IHC	Sigma-Aldrich	Cat # H1009
VECTASTAIN® ABC-HRP Kit, Peroxidase	IHC	Vector Labs	Cat # PK-6100
Vector® NovaRED® Substrate Kit, Peroxidase (HRP)	IHC	Vector Labs	Cat # SK-4800
Eukitt® Quick-hardening mounting medium	IHC	Sigma-Aldrich	Cat # 03989
DAPI	IF	Sigma-Aldrich	Cat # D9542
Rabbit anti-human CD8α	IF	Abcam	Cat # Ab4055
Rabbit anti-human CD103	IF	Abcam	Cat # Ab129202
Goat anti-rabbit AlexaFluor 647	IF	Invitrogen	Cat #A21245
Goat anti-rabbit AlexaFluor 546	IF	Invitrogen	Cat #A11035
Dako Fluorescence Mounting Medium	IF	Dako	Cat # S3023
Fetal Bovine Serum	IF	Sigma-Aldrich	Cat #F7524
TWEEN® 20	IF	Sigma-Aldrich	Cat # P1379
PAR-1 polyclonal antobody	IF	Bioss	Cat # bs-0828R
Rabbit anti-human MAP2	IF	ThermoFisher Scientific	Cat # PA5-17646
Mouse anti-human Nestin	IF	Sigma-Aldrich	Cat # MAB5326
Rabbit anti-mouse βIII-tubulin	IF	Cusabio	Cat # CSB-PA03874A0Rb
Rabbit anti-human NF-H	IF	Abcam	Cat # Ab8135
Rabbit anti-mouse CD8	IF	CellSignaling	Cat # D4W2Z
Biotinylated goat anti-rabbit	IF	Merck Millipore	Cat # SAB3700856
Anti-biotin streptavidin AlexaFluor 488	IF	Invitrogen	Cat # S11223
Goat anti-rabbit AlexaFluor 594	IF	Invitrogen	Cat # A11037
Mouse anti-mouse NeuN	IF	Sigma-Aldrich	Cat # MAB377
Rabbit anti-mouse AlexaFluor 680	IF	Invitrogen	Cat # A21065
Phospho-Tau (Ser202, Thr205) Monoclonal Antibody (AT8)	IF	ThermoFisher Scientific	Cat # MN1020
Phospho-Tau (Thr212, Ser214) Monoclonal Antibody (AT100)	IF	ThermoFisher Scientific	Cat # MN1060

Goat anti-mouse AlexaFluor 488	IF	Invitrogen	Cat # A-11001
A11 Anti-amyloid oligomer	IF	ThermoFisher Scientific	Cat # 57006
OC Anti-amyloid fibrils	IF	ThermoFisher Scientific	Cat # 57005
PBS 10X	Cell Isolation	Corning	Cat # 20-031-CV
HBSS 10X	Cell Isolation	ThermoFisher Scientific	Cat # 14025092
Adult Brain Dissociation kit	Cell Isolation	Miltenyi Biotech	Cat # 130-107-677
Non-neuronal biotin antibody cocktail	Cell Isolation	Miltenyi Biotech	Cat # 130-115-389
CD8 T cell isolation kit	Cell Isolation	Miltenyi Biotech	Cat # 130-104-075
Percoll®	Cell isolation	Merck Millipore	Cat # GE17-0891-01
Dextran from Leuconostoc spp.	Cell isolation	Sigma-Aldrich	Cat # 31392
Heparin bms	Cell isolation	BRISTOL-MYERS SQUIBB	AIC 13732019
Chromium Single Cell 3′ GEM, Library & Gel Bead Kit v3	Sequencing	10X Genomics	Cat # PN-1000092
Chromium NextGem Single Cell 3' GEM Library & Gel Bead kit v3.1, 4rx	Sequencing	10X Genomics	Cat # PN-1000128
Chromium Next GEM Chip G Single Cell kit, 16 rxn	Sequencing	10X Genomics	Cat # PN-1000127
NextSeq® 500 High Output v2.5 Kit (150 cycles)	Sequencing	Illumina	Cat # 20024907
Sucrose	Brain Storage	Sigma-Aldrich	Cat # 84100
Sodium azide	Brain Storage	Sigma-Aldrich	Cat # S8032
OCT gel (optimum cutting temperature)	Brain Storage	DDK Italia	Cat # 22-118
RIPA lysis and extraction buffer	Brain homogenates preparation	ThermoFisher Scientific	Cat # 89901
Immobilon Western Chemiluminescent HRP substrate	DotBlot	Merck Millipore	Cat # WBKLS0500
Nitrocellulose blotting membrane	DotBlot	Amersham	Cat # RPN303D
Bradford reagent, 5x concentrate	Protein quantification	SERVA	Cat # 39222.01
EasyPep Mini MS Sample prep Kit	Protein extraction	ThermoFisher Scientific	Cat # A40006
Pierce BCA Protein Assay Kit	Protein quantification	ThermoFisher Scientific	Cat # 23250
ELISA human amyloid beta 40	ELISA	ThermoFisher Scientific	Cat # KHB3481
ELISA human amyloid beta 42	ELISA	ThermoFisher Scientific	Cat # KHB3544
ELISA human Total Tau	ELISA	Prodotti Gianni	Cat # E1333Hu
ELISA human Tau pT231	ELISA	ThermoFisher Scientific	Cat # KHB8051
ELISA human Tau pS199	ELISA	ThermoFisher Scientific	Cat # KHB7041
ELISA human Tau pS396	ELISA	ThermoFisher Scientific	Cat # EEL188

FC = Flow Cytometry, IHC/IF= Immunohistochemistry/Immunofluorescence staining,

Supplementary Figure 7 | Dot blot of insoluble fibrils.

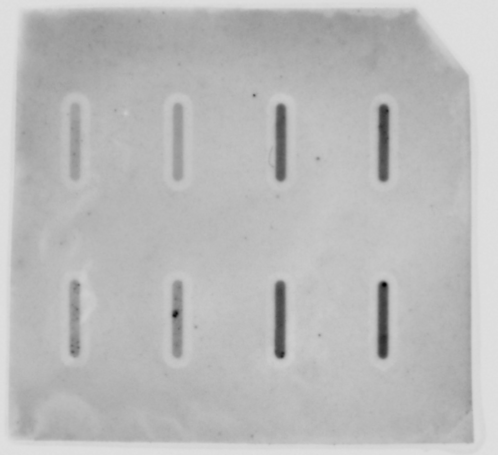

Columns 1 and 2: Isotype control; Columns 3 and 4: Anti-CD8α treatment.

Supplementary Figure 8 | Dot blot of insoluble oligomers.

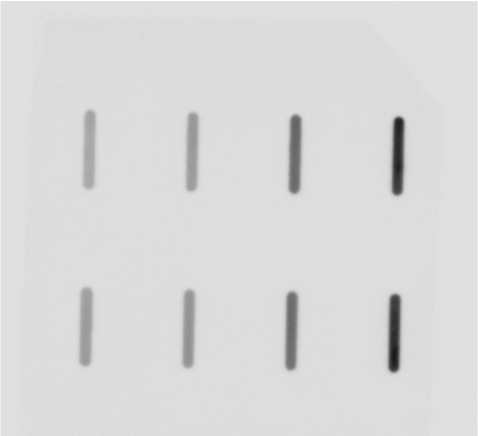

Columns 1 and 2: Isotype control; Columns 3 and 4: Anti-CD8α treatment.

Supplementary Figure 9 | Dot blot of soluble fibrils.

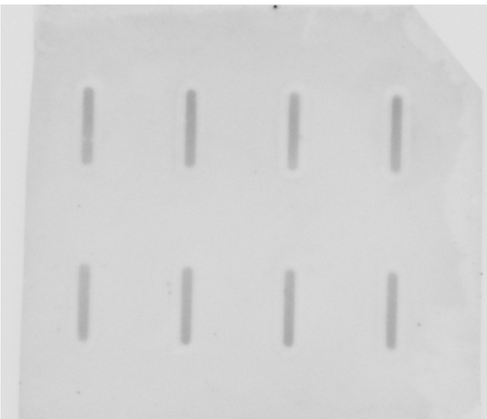

Columns 1 and 2: Isotype control; Columns 3 and 4: Anti-CD8α treatment.

Supplementary Figure 10 | Dot blot of soluble oligomers.

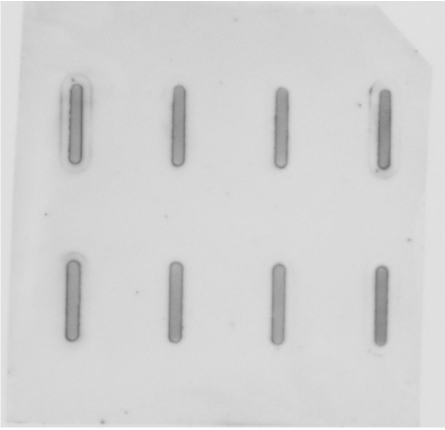

Columns 1 and 2: Isotype control; Columns 3 and 4: Anti-CD8α treatment.
