# Supplementary material for: CD103–CD8+ T cells promote neurotoxic inflammation in Alzheimer’s disease via granzyme K–PAR-1 signaling
Source: Nat Commun. 2025 Sep 24;16:8372. doi: 10.1038/s41467-025-62405-6 (PMC12460627; doi:10.1038/s41467-025-62405-6)
Supplement: Supplementary file 2 — Description of Additional Supplementary Files [file 41467_2025_62405_MOESM2_ESM.pdf]

Description of Additional Supplementary Files

Supplementary Data 1, scRNAseq mouse brain and meninges

Supplementary Data 2, scRNAseq human blood

Supplementary Data 3, scRNAseq human CSF

Supplementary Data 4, Proteomic experiment

Supplementary Movie 1- Ctrl-. It is the Ctrl- for neurons cultured in presence of GrK. It shows intraneuronal Ca^{2+} release in not-treated neurons.

Supplementary Movie 2- GrK 150 nM. It shows intraneuronal Ca^{2+} release induced by GrK treatment.

Supplementary Movie 3- GrK 150 nM + SCH79797 100 nM. It shows intraneuronal Ca^{2+} release induced by GrK treatment in presence of SCH79797 PAR-1 inhibitor.
