# Supplementary material for: CD103–CD8+ T cells promote neurotoxic inflammation in Alzheimer’s disease via granzyme K–PAR-1 signaling
Source: Nat Commun. 2025 Sep 24;16:8372. doi: 10.1038/s41467-025-62405-6 (PMC12460627; doi:10.1038/s41467-025-62405-6)
Supplement: Supplementary file 10 — Reporting Summary [file 41467_2025_62405_MOESM10_ESM.pdf]

nature portfolio

Corresponding author(s): Gabriela Constantin

Last updated by author(s): 27/06/2025

Reporting Summary

Nature Portfolio wishes to improve the reproducibility of the work that we publish. This form provides structure for consistency and transparency in reporting. For further information on Nature Portfolio policies, see our Editorial Policies and the Editorial Policy Checklist.

Statistics

For all statistical analyses, confirm that the following items are present in the figure legend, table legend, main text, or Methods section.

n/a	Confirmed	
☐	☒	The exact sample size (*n*) for each experimental group/condition, given as a discrete number and unit of measurement
☐	☒	A statement on whether measurements were taken from distinct samples or whether the same sample was measured repeatedly
☐	☒	The statistical test(s) used AND whether they are one- or two-sided<br>*Only common tests should be described solely by name; describe more complex techniques in the Methods section.*
☐	☒	A description of all covariates tested
☐	☒	A description of any assumptions or corrections, such as tests of normality and adjustment for multiple comparisons
☐	☒	A full description of the statistical parameters including central tendency (e.g. means) or other basic estimates (e.g. regression coefficient) AND variation (e.g. standard deviation) or associated estimates of uncertainty (e.g. confidence intervals)
☒	☐	For null hypothesis testing, the test statistic (e.g. *F*, *t*, *r*) with confidence intervals, effect sizes, degrees of freedom and *P* value noted<br>*Give P values as exact values whenever suitable.*
☒	☐	For Bayesian analysis, information on the choice of priors and Markov chain Monte Carlo settings
☐	☒	For hierarchical and complex designs, identification of the appropriate level for tests and full reporting of outcomes
☐	☒	Estimates of effect sizes (e.g. Cohen's *d*, Pearson's *r*), indicating how they were calculated

Our web collection on statistics for biologists contains articles on many of the points above.

Software and code

Policy information about availability of computer code

Data collection	Raw base call (BCL) files were processed using Cell Ranger v 6.0.1.<br>The images were taken using Zeiss software ZEN.<br>FACS data were collected by BD FACS Diva Software V9.
Data analysis	SingleCellExperiment object was uploaded and analyzed using PartekFlow analysis software.<br>Images were analyzed using ZenBlue software (ZEISS).<br>Statistical analysis were done by Prism V9.<br>FACS data were analyzed by FlowJo v10.0.<br>Raw mass spectrometry data were processed using Proteome Discoverer (v2.5), and then analyzed using PathFinder and SimplyEnrichment R packages, which codes are yet available in GitHub.

For manuscripts utilizing custom algorithms or software that are central to the research but not yet described in published literature, software must be made available to editors and reviewers. We strongly encourage code deposition in a community repository (e.g. GitHub). See the Nature Portfolio guidelines for submitting code & software for further information.

Data

Policy information about availability of data

All manuscripts must include a data availability statement. This statement should provide the following information, where applicable:

- Accession codes, unique identifiers, or web links for publicly available datasets
- A description of any restrictions on data availability
- For clinical datasets or third party data, please ensure that the statement adheres to our policy

The data that support the findings of this study are available in this manuscript and supplementary informations.
Single cell RNA sequencing dataset of PBMCs from HCs and AD human patients published by Xu, et al 2021, is available under the accession code GSE181279 (https://www.ncbi.nlm.nih.gov/geo/query/acc.cgi?acc=GSE181279).
Single cell RNA sequencing dataset of CD45+ cells isolated from the CSF of HCs and AD and MCI human patients published by Gate et al 2020, is available under the accession code GSE134578 (https://www.ncbi.nlm.nih.gov/geo/query/acc.cgi?acc=GSE134578).
Our single cell RNA sequencing dataset of CD45HIGH leukocyes isolated from the brain and meninges of 6 months old WT and 3xTg-AD mice is available under the accession codes GSE180188 (https://www.ncbi.nlm.nih.gov/geo/query/acc.cgi?acc=GSE180188) and GSE180184 (https://www.ncbi.nlm.nih.gov/geo/query/acc.cgi?acc=GSE180184).
The mass spectrometry proteomics data have been deposited to the ProteomeXchange Consortium via the PRIDE partner repository with the dataset identifier PXD064640 (https://www.ebi.ac.uk/pride/archive/projects/PXD064640)
All data associated with this study can be found in the paper or in supplementary materials. Raw data generated in this study are provide in the Supplementary Information and Source Data files

Research involving human participants, their data, or biological material

Policy information about studies with human participants or human data. See also policy information about sex, gender (identity/presentation), and sexual orientation and race, ethnicity and racism.

Reporting on sex and gender	We considered only female control subjects and AD patients, as described in Table 1.
Reporting on race, ethnicity, or other socially relevant groupings	No informations about race or ethnicity is provided.
Population characteristics	Group characteristics, including sex and age for each patient, are presented in Table 1. AD patients and control subjects considered in the study were age- and sex-matched.
Recruitment	Formalin-fixed paraffin-embedded (FFPE) hippocampal sections of controls and Alzheimer's disease (AD) cases were obtained from the Medical Research Council (MRC) London Neurodegenerative Disease Brain Bank.
Ethics oversight	Written informed consent for brain autopsy had been obtained by MRC London Neurodegenerative Disease Brain Bank. The neuropathology studies were approved by the Ethical Committee of the University of Verona (protocol nr. 20794).

Note that full information on the approval of the study protocol must also be provided in the manuscript.

Field-specific reporting

Please select the one below that is the best fit for your research. If you are not sure, read the appropriate sections before making your selection.

☒ Life sciences ☐ Behavioural & social sciences ☐ Ecological, evolutionary & environmental sciences

For a reference copy of the document with all sections, see nature.com/documents/nr-reporting-summary-flat.pdf

Life sciences study design

All studies must disclose on these points even when the disclosure is negative.

Sample size	Sample size was choosen based on prior publications using human and mouse samples, which provided estimations od expected effect sizes and variance (see PMID: 26214837)
Data exclusions	For Y-Maze test, animals with no movement more than 30% of the whole time of the test were excluded from the analysis and values were removed with the "Identify outliers" function of Prism 9(Method ROUT, Q = 10 %)<br>For CFC test mice with freezing time less than 30% of the average of the freezing time in each group have been excluded from the analysis.<br>For FACS, ELISA, DotBlot and immunohistochemistry analysis, were excluded from the analysis values with the "Identify outliers" function of Prism 9 (Method ROUT, Q = 10 %).
Replication	All data were successfully replicated in at least two biological replicates and in at least two independendent experiments.
Randomization	All mice were randomly assigned to the experimental groups. For human samples, brain tissues were allocated to groups considering characteristics presented in Table 1, especially age and sex, trying to consider an homogeneous group of patients, thus reducing the

variability.

Blinding

Investigators were blinded with respect to the conditions for neuropathological, behavioral, scRNAseq, and flow cytometry studies. Particularly, samples were coded during the experiments, making unpossible to refer the sample to a condition or treatment.
For human samples, samples were selected and coded making unpossible to refer the sample to a condition during the experiment.
For experiments with cell lines or primary neurons, conditions were coded making unpossible to refer them to a specific condition during the experiment.
Samples were decoded only after experiments and analyses.

Reporting for specific materials, systems and methods

We require information from authors about some types of materials, experimental systems and methods used in many studies. Here, indicate whether each material, system or method listed is relevant to your study. If you are not sure if a list item applies to your research, read the appropriate section before selecting a response.

Materials & experimental systems

n/a	Involved in the study	
	x	Antibodies
	x	Eukaryotic cell lines
x		Palaeontology and archaeology
	x	Animals and other organisms
x		Clinical data
x		Dual use research of concern
x		Plants

Methods

n/a	Involved in the study	
x		ChIP-seq
	x	Flow cytometry
x		MRI-based neuroimaging

Antibodies

Antibodies used

As described in Table 2 of the supplementary material, the following antibodies have been used:
Anti-CD16/32 Fc-Block FC BioLegend Cat # 101302; 1:200
Anti-CD11a/CD18 FITC FC Miltenyi Biotech Cat # 130-114-422; 1:50
Anti-CD103 BV421 FC BD Bioscences Cat # 562771; 1:50
Anti-CD8 APC-H7 FC BD Bioscences Cat # 560182; 1:50
Anti-CD69 BV650 FC BD Bioscences Cat # 740460; 1:50
Anti-TCRgd PE-CF594 FC BD Bioscences Cat # 563532; 1:50
Anti-CD62L PE FC BD Bioscences Cat # 553151; 1:50
Anti-CD45 BV605 FC BD Bioscences Cat # 563053; 1:50
Anti-CD27 APC FC BD Bioscences Cat # 560961; 1:50
Anti-CD11b APC-R700 FC BD Bioscences Cat # 564985; 1:50
Anti-Ly6g BV510 FC BD Bioscences Cat # 740157; 1:50
Anti-CD4 PE-Cy7 FC BD Bioscences Cat # 552775; 1:50
Anti-CD197 (CCR7) BV786 FC BD Bioscences Cat # 564355; 1:50
Anti-CD44 BV711 FC BD Bioscences Cat # 563971; 1:50
Viobility TM Fixable Dye 405/520 FC Miltenyi Biotech Cat # 130-109-814; 1:50
Anti-CD45 APC-Vio770 FC Miltenyi Biotech Cat # 130-110-662; 1:100
Anti-CD8 PE-Cy7 FC Biolegend Cat # 100722; 1:100
Anti-CD3 BV650 FC BD Bioscences Cat # 564378; 1:100
Rabbit anti-mouse/human GrK FC/IF ThermoFisher Scientific Cat # PA550980; 1:100
Goat anti-rabbit AlexaFluor 488 FC/IF ThermoFisher Scientific Cat # A11034; 1:100
Anti-VLA-4 (CD49d) PE-Cy7 FC BioLegend Cat # 103618; 1:50
Anti-Ly6g BV421 FC BD Bioscences Cat # 562737; 1:50
Anti-CD44 BV510 FC BD Bioscences Cat # 563114; 1:50
Anti-CD45 BV786 FC BD Bioscences Cat # 564225; 1:50
Anti-CD4 APC FC BD Bioscences Cat # 553051; 1:50
Anti-CD45 BV480 FC BD Bioscences Cat # 566095; 1:50
Anti-KLRG1 FC BD Bioscences Cat # 561621; 1:50
Anti-Ras isotype antibody In Vivo Treatment Produced in house Produced in house; 0.22 mg/mouse
Anti-mouse CD8a In Vivo Treatment BioXCell BE0061; 0.22 mg/mouse
Anti-β-Amyloid, 1-16 Antibody (6E10) IHC BioLegend Cat# SIG-39320; 1:1000
Tau Monoclonal Antibody (HT7) IHC/IF ThermoFisher Scientific Cat # MN1000; 1:200
Phospho-Tau (Thr231) Monoclonal Antibody (AT180) IHC/IF ThermoFisher Scientific Cat # MN1040; 1:200
DAPI IF Sigma-Aldrich Cat # D9542; 1:1000
Rabbit anti-human CD8a IF Abcam Cat # Ab4055; 1:100
Rabbit anti-human CD103 IF Abcam Cat # Ab129202; 1:100
Goat anti-rabbit AlexaFluor 647 IF Invitrogen Cat #A21245; 1:1000
Goat anti-rabbit AlexaFluor 546 IF Invitrogen Cat #A11035; 1:1000

PAR-1 polyclonal antobody IF Bioss Cat # bs-0828R; 1:100
Anti-GrA eFluor450 FC ThermoFisher Scientific Cat # 48-5831-82; 1:50
Anti-CD69 BV605 FC BD Bioscences Cat # 563290; 1:50
Anti-TCRgd BV650 FC ThermoFisher Scientific Cat # 416-5711-82; 0.75:50
Anti-CD103 BV711 FC ThermoFisher Scientific Cat # 407-1031-82; 2.550
Anti-CD45 BV786 FC ThermoFisher Scientific Cat # 417-0451-82; 2.5:50
Anti-GrB PE FC SONY Cat # 2461035; 1:.7550
Anti-CD4 Pe-Cy7 FC ThermoFisher Scientific Cat # 25-0041-82; 1.5:50
Anti-Eomes eFluor660 FC ThermoFisher Scientific Cat # 50-4875-82; 2.5:50
Anti-CD8 APC-AF750 FC ThermoFisher Scientific Cat # MCD0827; 4:50
Anti-CD69 SB436 FC ThermoFisher Scientific Cat # 62-0691-82; 1:50
Anti-CXCR3 FC ThermoFisher Scientific Cat # 63-1831-82; 2:50
Anti-CD45 BV711 FC ThermoFisher Scientific Cat # 407-0451-82; 1:50
Anti-CX3CR1 FITC FC ThermoFisher Scientific Cat # ACR-059-F; 1.5:50
Anti-CD103 PE FC ThermoFisher Scientific Cat # 12-1031-82; 1.5:50
Anti-CD62L PE-TexasRed FC ThermoFisher Scientific Cat # RM4317; 2.5:50
Anti-CXCR6 APC FC ThermoFisher Scientific Cat # 17-9186-82; 2.5:50
A11 Anti-amyloid oligomer IF ThermoFisher Scientific Cat # 57006; 1:100
OC Anti-amyloid fibrils IF ThermoFisher Scientific Cat # 57005; 1:100
Rabbit anti-human MAP2 IF ThermoFisher Scientific Cat # PA5-17646; 1:100
Mouse anti-human Nestin IF Sigma-Aldrich Cat # MAB5326; 1:100
Rabbit anti-mouse bIII-tubulin IF Cusabio Cat # CSB-PA03874A0Rb; 1:100
Rabbit anti-human NF-H IF Abcam Cat # Ab8135; 1:100
Rabbit anti-mouse CD8 IF CellSignaling Cat # D4W2Z; 1:100
Biotinylated goat anti-rabbit IF Merck Millipore Cat # SAB3700856; 1:1000
Anti-biotin streptavidin AlexaFluor 488 IF Invitrogen Cat # S11223; 1:1000
Goat anti-rabbit AlexaFluor 594 IF Invitrogen Cat # A11037; 1:1000
Mouse anti-mouse NeuN IF Sigma-Aldrich Cat # MAB377; 1:200
Rabbit anti-mouse AlexaFluor 680 IF Invitrogen Cat # A21065; 1:1000
Phospho-Tau (Ser202, Thr205) Monoclonal Antibody (AT8) IF ThermoFisher Scientific Cat # MN1020; 1:1000
Phospho-Tau (Thr212, Ser214) Monoclonal Antibody (AT100) IF ThermoFisher Scientific Cat # MN1060; 1:1000
Goat anti-mouse AlexaFluor 488 IF Invitrogen Cat # A-11001; 1:100

Validation

Validation data for all commercial antobodies for species and applications are available on the manufacturers websites. Each primary antibody was validated using proper fluorophore controls and positive signal was evaluated versus an unstained sample. In house produced anti-Ras antibody for in vivo treatment was previously used by our group and data were published (see PMID: 26214837). Anti-mouse GrK primary antibody was tested and validated for flow cytometry and the results are availavle in the supplementary material of the present work.

Eukaryotic cell lines

Policy information about cell lines and Sex and Gender in Research

Cell line source(s)

Primary neurons were isolated from new-born 3xTg-AD mice as described in the Method section. Primary CD8+ T cells were isolated from the liver of 3xTg-AD mice and sorted as described in the Method section. Commercially available human neuroblastoma SH-SY5Y cells were cultured as described in the Method section and obtained form Sigma-Merk (94030304-CDNA-20UL).

Authentication

Cell lines were not authenticated

Mycoplasma contamination

All cell lines were tested and were negative for mycoplasma contamination.

Commonly misidentified lines
(See ICLAC register)

No misidentifies lines were used.

Animals and other research organisms

Policy information about studies involving animals; ARRIVE guidelines recommended for reporting animal research, and Sex and Gender in Research

Laboratory animals	3xTg-AD (MMRRC stock no. 34830-JAX), Itgal-/- (stock no. 005257) and WT B6129SF2/J (stock no. 101045) mice were purchased from the Jackson Laboratory. 3xTg-AD mice harbor human mutations for APP, PSEN1 and TAU proteins, developing both amyloid and tau pathologies. We backcrossed 3xTg-AD and Itgal-/- mice to obtain a transgenic line with all transgenes from the 3xTg-AD and Itgal-/- models (APPSwe, tauP301L, PS1M146V knock-in and LFA-1 knockout).<br><br>All mice were housed in pathogen-free climate-controlled facilities with a 12 hours dark/light cycle, and were provided with food and water ad libitum.<br><br>For experiments we used 6 months old mice.
Wild animals	No wild animal used in this study.
Reporting on sex	Both male and female mice were used in this study.
Field-collected samples	Blood samples were collected from the retro-orbital plexus of anesthetized mice using sodium heparinized capillaries.
Ethics oversight	The research conducted in this study complies with all relevant ethical guidelines. Research involving animals was authorized by the Ethical Committee from the University of Verona and by the Italian Ministry of Health, Department of Veterinary Public Health, Nutrition and Food Safety, Directorate General of Animal Health and Veterinary Medicine (authorization no. 876/2021-PR), as required by Italian legislation (D. Lgs 26/2014) as per the application of European Directive (2010/63/UE). All efforts were made to minimize the number of animals used and their suffering during the experimental procedures.

Note that full information on the approval of the study protocol must also be provided in the manuscript.

Plants

Seed stocks	No plant was used in the study.
Novel plant genotypes	No plant was used in the study.
Authentication	No plant was used in the study.

Flow Cytometry

Plots

Confirm that:

- [x] The axis labels state the marker and fluorochrome used (e.g. CD4-FITC).
- [x] The axis scales are clearly visible. Include numbers along axes only for bottom left plot of group (a 'group' is an analysis of identical markers).
- [x] All plots are contour plots with outliers or pseudocolor plots.
- [x] A numerical value for number of cells or percentage (with statistics) is provided.

Methodology

Sample preparation	Preparation of single-cell suspensions<br>Meninges<br>Dura mater and leptomeninges were carefully removed from the interior aspect of skull and surfaces of brains with fine surgical curved scissors and forceps, and enzymatically digested with a collagenase/DNase I solution (collagenase crude type IA, Merck Millipore; Deoxiribonuclease I crude lyophilized, Merck Millipore) at 37 °C for 15 min. Cells were washed with cold PBS 1X.<br>Brain<br>Brains were collected in cold PBS1X and separated from leptomeninges. They were homogenized using a gentleMACS Dissociator (Miltenyi Biotec), and enzymatically digested with a collagenase/DNase I solution (collagenase crude type IA, Merck Millipore; Deoxiribonuclease I crude lyophilized, Merck Millipore) at 37 °C for 45 minutes. Cells were passed through a 70-µm cell strainer into a new tube for Percoll (Merck Millipore) gradient centrifugation, and cells recovered from the interphase were washed with cold PBS 1X.

Brain and meninges
Brains were collected without peeling the meninges in cold PBS1X. After choroid plexus removal, brains were homogenized using a gentleMACS Dissociator (Miltenyi Biotec), and enzymatically digested with a collagenase/DNase I solution (collagenase crude type IA, Merck Millipore; Deoxiribonuclease I crude lyophilized, Merck Millipore) at 37 °C for 45 minutes. Cells were passed through a 70-µm cell strainer into a new tube for Percoll (Merck Millipore) gradient centrifugation, and cells recovered from the interphase were washed with cold PBS 1X.
Blood
Blood samples were collected from the retro-orbital plexus of anesthetized mice using sodium heparinized capillaries and were mixed with an equal volume of 1% dextran from Leuconostoc spp (Sigma-Aldrich) plus 10 U/ml sodium heparin. After pelleting the erythrocytes, the overlying supernatant plasma/dextran suspension of leukocytes was washed in cold PBS 1X.
Spleen
Spleens were mechanically disrupted, and single-cell suspensions were obtained by passing the cells through a 70-µm strainer. After erythrocyte lysis by NaCl 0.2% and 1.2%, cells were washed with cold PBS 1X.
Liver
Livers were collected in cold RPMI culture medium (Corning). They were mechanically homogenized firstly using a scalpel and then using a gentleMACS Dissociator (Miltenyi Biotec). Then, they were enzymatically digested with a collagenase/DNase I solution at 37 °C for 30 min. Cells were passed through a 70-µm cell strainer into a new tube for Percoll (Merk Millipore) gradient centrifugation, and cells recovered from the interphase were washed with cold PBS 1X.

Instrument

BD FACSAria Fusion; BD LSRFortessa.

Software

Data Collection: BD FACSDiva.

Data analysis: FlowJo v10

Cell population abundance

As reported in gating strategies shown in Extended Fig. 5:

Brain: after the removal of doublets and death cells, we obtained around the 10.2% of CD45high cells (leukocytes). Of them, around 5.91% were neutrophils, around 20.2% were myeloid cells, and around the 72.5% were CD11b-Ly6g- cells. From the CD11b-Ly6g- population we detected around the 16.5% of CD8+ T cells, which were analysed for the expression of CD103 and CD69 surface markers. In a separate panel, it was evaluated the expression of GrK on these cells subsets.

Meninges: after the removal of doublets and death cells, we obtained around the 61.8% of CD45+ cells (leukocytes). Of them, around 9.07% were neutrophils, around 59% were myeloid cells, and around the 31.8% were CD11b-Ly6g- cells. From the CD11b-Ly6g- population we detected around the 35.8% of CD8+ T cells, which were analysed for the expression of CD103 and CD69 surface markers. In a separate panel, it was evaluated the expression of GrK on these cells subsets.

Spleen: after the removal of doublets and death cells, we obtained around the 99.6% of CD45+ cells (leukocytes). Of them, around 4.89% were neutrophils, around 4.9% were myeloid cells, and around the 88.4% were CD11b-Ly6g- cells. From the CD11b-Ly6g- population we analysed the percentages of CD8+ T cells.

Blood: after the removal of doublets and death cells, we obtained around the 99.8% of CD45+ cells (leukocytes). Of them, around the 90.7% were CD11b-Ly6g- cells. From the CD11b-Ly6g- population we detected around the 35.9% of CD8+ T cells, which were analysed for the expression of CD44, KLRG1, and LFA-1 surface markers. In a separate panel, it was evaluated the expression of GrK on these cells subsets.

Cell sorting from the liver: after the removal of doublets and death cells, we obtained around the 54.1% of CD8+ T cells, of them, around the 40.6% expressed CD69 surface marker. From the CD8+ CD69+ population we sorted CD103+ cells (around the 12.8%) and CD103- cells (around the 87.2%). After sorting cells were fixed, purity and GrK expression were checked by flow cytometry and immunofluorescent staining. Rapresentative images are shown in main figures.

Gating strategy

Gating strategies are shown in Extended Fig. 5.
For all the samples, we morphologically identifed lymphocytes. Next, we removed doublets and death cells by using 7AAD viobility marker. Then CD45high (brain)) or CD45+ (meninges, blood, spleen, liver) were gated. From them, CD11b+Ly6g+ neutrophils were identified. On the contrary, the CD11b-Ly6g- population was used as starting point for the identification of CD4+ or CD8+, or gd T cells. Next, we identified CD69+ CD103+ and CD69+CD103- Trm cell subsets from the CD8+ population. On them the expression of intracellular and extracellular molecules was evaluated. Cell abundances and molecule expressions were evaluated in separated experients using separated panels, as described in the Material&Methods section of the present work.

☒ Tick this box to confirm that a figure exemplifying the gating strategy is provided in the Supplementary Information.
